# Monitoring Ambient Parameters in the IoT Precision Agriculture Scenario: An Approach to Sensor Selection and Hydroponic Saffron Cultivation

**DOI:** 10.3390/s22228905

**Published:** 2022-11-17

**Authors:** Kanwalpreet Kour, Deepali Gupta, Kamali Gupta, Divya Anand, Dalia H. Elkamchouchi, Cristina Mazas Pérez-Oleaga, Muhammad Ibrahim, Nitin Goyal

**Affiliations:** 1 Chitkara University Institute of Engineering & Technology, Chitkara University, Rajpura 140401, Punjab, India; 2 School of Computer Science and Engineering, Lovely Professional University, Phagwara 144411, Punjab, India; 3 Higher Polytechnic School, Universidad Europea del Atlántico, C/Isabel Torres 21, 39011 Santander, Spain; 4 Department of Information Technology, College of Computer and Information Sciences, Princess Nourah bint Abdulrahman University, P.O. Box 84428, Riyadh 11671, Saudi Arabia; 5 Research and Innovation, Universidad Europea del Atlántico, C/Isabel Torres 21, 39011 Santander, Spain; 6 Department of Project Management, Universidade Internacional do Cuanza, Estrada Nacional 250, Bairro Kaluapanda, Cuito-Bié P.O. Box 841, Angola; 7 Department of Project Management, Universidad Internacional Iberoamericana, Campeche 24560, Mexico; 8 Department of Computer Engineering and Research Center of Advance Technology, Jeju National University, Jeju-si 63243, Republic of Korea; 9 Department of Computer Science and Engineering, Central University of Haryana, Mahenderagarh 123031, Haryana, India

**Keywords:** IoT, saffron, sensors, precision agriculture, smart farming, hydroponics, NFT

## Abstract

The world population is on the rise, which demands higher food production. The reduction in the amount of land under cultivation due to urbanization makes this more challenging. The solution to this problem lies in the artificial cultivation of crops. IoT and sensors play an important role in optimizing the artificial cultivation of crops. The selection of sensors is important in order to ensure a better quality and yield in an automated artificial environment. There are many challenges involved in selecting sensors due to the highly competitive market. This paper provides a novel approach to sensor selection for saffron cultivation in an IoT-based environment. The crop used in this study is saffron due to the reason that much less research has been conducted on its hydroponic cultivation using sensors and its huge economic impact. A detailed hardware-based framework, the growth cycle of the crop, along with all the sensors, and the block layout used for saffron cultivation in a hydroponic medium are provided. The important parameters for a hydroponic medium, such as the concentration of nutrients and flow rate required, are discussed in detail. This paper is the first of its kind to explain the sensor configurations, performance metrics, and sensor-based saffron cultivation model. The paper discusses different metrics related to the selection, use and role of sensors in different IoT-based saffron cultivation practices. A smart hydroponic setup for saffron cultivation is proposed. The results of the model are evaluated using the AquaCrop simulator. The simulator is used to evaluate the value of performance metrics such as the yield, harvest index, water productivity, and biomass. The values obtained provide better results as compared to natural cultivation.

## 1. Introduction

**Figure 1:**
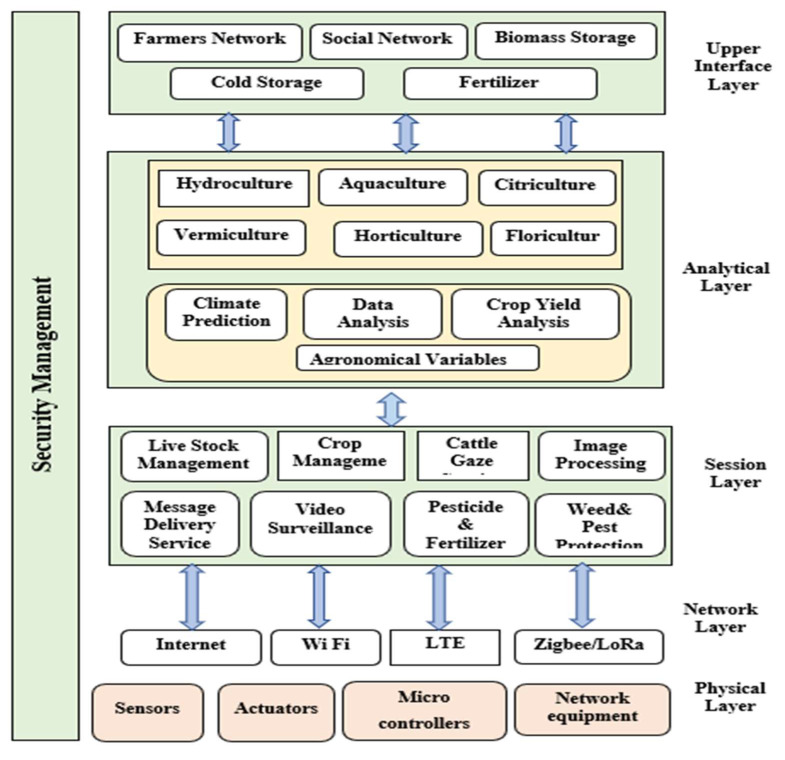
Smart Agricultural Framework using IoT.

The exponential increase in the population, which will reach 9.8 billion by year 2050, will have its maximum impact on developing nations such as India [1]. This will lead to an increase in the demand for food. Traditional methods of cultivation and the deficit of land available for agriculture place limits on the yields obtained from agriculture. To minimize the demand and supply gap, in light of the increasing urbanization trend, with approximately 70% of the world population estimated to be urbanized by 2050, there is a need for new, innovative and sustainable solutions in agriculture to meet the increasing needs for agricultural products [1]. This can be achieved using IoT with multifarious other technologies. IoT may be defined as the network of ‘things’ that collect, save, and analyze data. Sensors are the backbone of IoT networks. Sensors may be defined as small devices based on physical principles of origin that detect any event, convert the received stimulus and transmit a resulting impulse [2,3]. Sensors form an integral part of all the IoT applications, including precision agriculture. The use of sensors in smart farming can be clearly seen in Figure 1 in a physical layer which includes areas such as farms, hydroponics, greenhouses, livestock management, smart irrigation, transportation, aerial man vehicles, etc. Using IoT, the data collected by sensors are wirelessly sent to the cloud server, which can be accessed from anywhere using an internet-enabled device [3].

The internet of things has enhanced the potential of all sectors of life, including agriculture. Various innovative agricultural practices and the presence of different sensors for all kinds of agricultural practices, such as pest detection, irrigation, etc., are evolving day by day, increasing the productivity and rendering tasks easier [4]. According to different reports, the population of the world will increase by 25 percent of the present value and reach approximately 10 billion. In order to accommodate this change, there is a great need for increases in agricultural efficiency and productivity [5]. The evolution of smart farming, or precision agriculture, in the last decade has led to better decision making through the collection and analysis of data. As shown in Figure 1, smart agriculture consists of different layers, with a user interface layer for providing the applications [6]. The detailed architecture of the IoT in relevance to agriculture is shown in Figure 1.

Agricultural sensors have improved data analysis to a great extent by providing the most accurate data with very low error percentages. This has led to increased productivity, a lack of degradation of natural resources, easy supply chain management, and proper water resource usage. The selection of sensors for a specific environment and crop’s growth is crucial for accurate prediction and performance in smart farming. Using sensors, the farmers can measure properties such as the temperature, moisture, humidity, pH, soil properties, etc., resulting in the maximization of the yield and quality of crops [7,8]. The performance of sensor data generation depends on many factors, such as the node placement and network performance [9].

Sensor data are collected and analyzed by wireless transmission through the network to the cloud, allowing the user to access the analyzed data and control the farm or field environment remotely. However, there are some complications related to the use and selection of sensors in developing nations such as India, which include their high cost, feasibility, reliability, the time consumed during their implementation, etc. Additional complications also include Wi-Fi availability, the customization of sensors for a particular environment, error handling, and fault tolerance [9].

**Figure 2:**
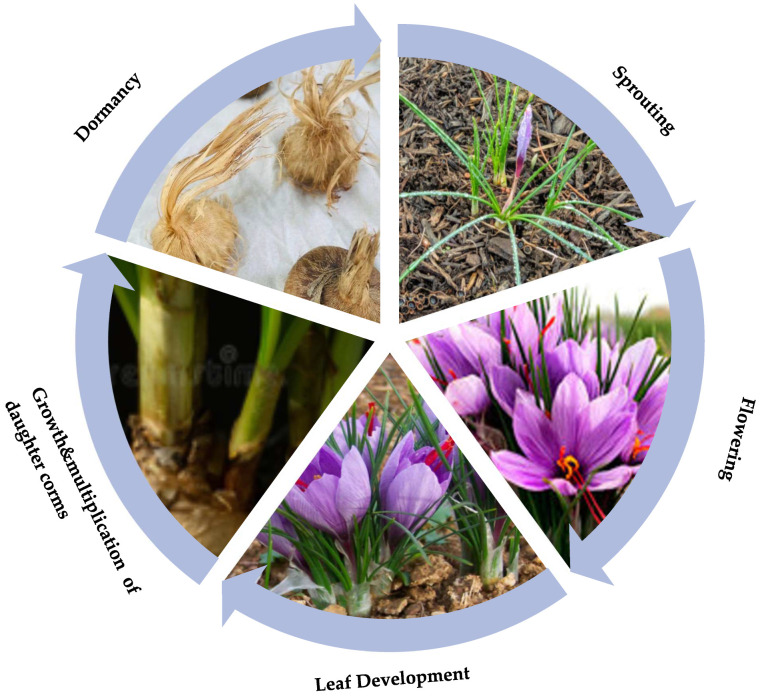
Growth Cycle of Saffron.

The potential of saffron cultivation in India can be increased by propagating it by artificial means using IoT sensors [3,5,10]. The conditions and the sensor configurations that are used for artificial and natural media are different [5,10]. Although researchers have worked on artificial saffron cultivation, there is a lack of studies related to the criteria for choosing sensors, a proper model for growth, and detailed sensor configurations that can be used in artificial environments. The vegetative growth cycle of Saffron can be seen in Figure 2.

The availability of different sensors is shown in Figure 2, since monitoring a common parameter also creates a great deal of chaos. In order to solve this problem, all the parameters related to sensor selection, such as the range, sensitivity, error, resolution, precision, etc., need to be minutely analyzed [11]. 

This paper is the first of its kind to emphasize sensor selection criteria for IoT-based saffron cultivation. The paper highlights the sensors most frequently used in automated environments and the criteria for choosing the suitable ones as per the environment. The outcomes of this research could be of great value and benefit to new researchers who are working on developing an IoT-based automated framework for saffron cultivation. The major contributions of this paper are as follows:

- A detailed analysis of the sensors required for artificial saffron cultivation and the factors motivating the selection of sensors in PA (precision agriculture).
- An experimental setup for the hydroponic cultivation of saffron with different sensors, devices, and controlled agronomical variables.
- The implementation of the system model and block diagram, consisting of sensors deployed on the basis of the study of the hydroponic growth of saffron.
- An evaluation of the designed system model using AquaCrop Simulator for a comparison between the different output parameters.

**Figure 3:**
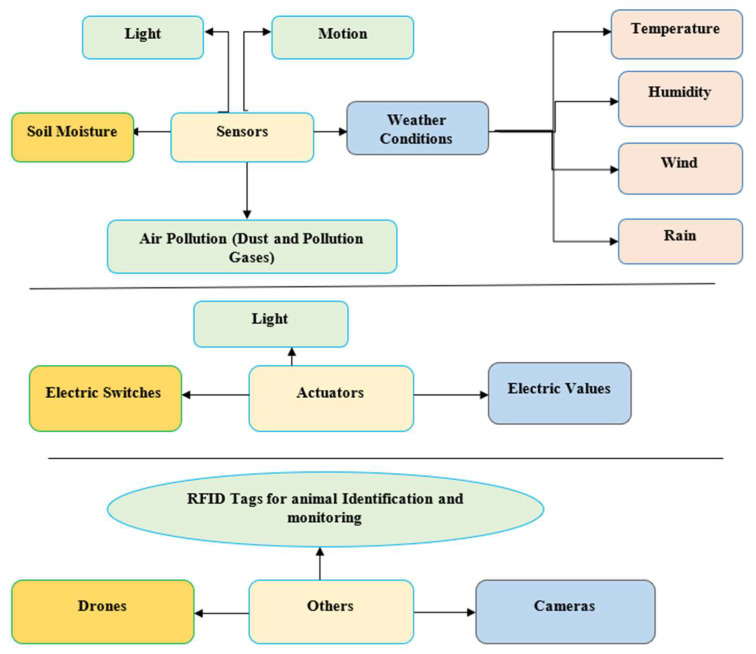
Sensor Controlled Parameters in Smart Agriculture.

The boom of IoT has led the sensor manufacturing industry to blossom, earning a profit of 4 billion USD annually. The availability of different sensors for a single parameter, as shown in Figure 3, has led to a great deal of chaos in the process of choosing a sensor for analyzing data in a particular environment. As a result, a wide spectrum of sensors are used, such as temperature sensors, motion sensors, proximity sensors, pressure sensors, position sensors, etc. [2,4,12]. The plant chosen for this study is saffron due to its rarity and expensiveness on the market [13]. Saffron or ‘red gold’ is derived from the stigma of the Crocus Sativus plant. It is propagated only by vegetative means using corms. The vegetative cycle of saffron lasts for about 240 days (approx.) and involves five major phases: sprouting (September–October), flowering (October–November), leaf development (October–April), the growth and multiplication of daughter corms (November–April), and dormancy (May–August) [10]. In Figure 3, sensors used for monitoring different agriculture parameters such as the soil moisture, luminosity, and humidity, among others, are shown.

The structure of the paper is arranged as follows: Section 2 presents a detailed analysis of all the factors involved in sensor selection and discuss the sensors used by different researchers, to date, in smart farming. Section 3 illustrates the research problems related to sensor selection and the factors to be considered. Section 4 outlines the major factors to be considered when selecting sensors and discusses the proposed framework and the status of the hydroponic cultivation of saffron using the designed setup. Section 5 shows the results from AquaCrop and provides an analysis of the different research questions discussed in Section 3. Section 6 explains the future prospects of the developed smart system, with concluding remarks.

## 2. Related Work

The work performed by researchers in precision agriculture can be discussed under two major headings. The former deals with all the different kinds of sensors used in the cultivation of different kinds of crops in diverse media. The important factors affecting the cultivation of crops are also discussed. The research conducted thus far and the sensors most commonly used for five major agronomical factors of saffron are discussed, along with their related characteristics, in the form of Table 1 and Table 2.

### 2.1. Sensors Used in Precision Farming

Different researchers have worked on the IoT implementation and applications in the agricultural sector. Starting from the year 2003, the number of publications related to precision agriculture have increased significantly. However, there are different dimensions of precision agriculture which have not been addressed. Most of the research on the use of IoT for agriculture is based on industrial crops, with much less attention devoted to economically potent but rare crops such as saffron [14]. There are far less research contributions regarding the sensor configurations used for artificial saffron cultivation [15].

In [16], the authors studied the use of soil moisture sensors to measure the water content of soil. It was found that there are various sensors, such as granular matrix sensors, VH400 sensors, and tensiometers for soil moisture measurement on the market, varying in their performance, accuracy, power consumption, cost, and the type of soil. The cost was found to be the most important factor influencing the selection of sensors.

In [17], the authors proposed a low-cost precision agriculture network using Lora WAN by utilizing low-cost sensors for soil water content monitoring and comparing the results obtained with a commercial sensor from Sentek. The results were compared in different soils, such as silty and loamy soils. The results obtained indicated the satisfactory accuracy of the moisture measurements; however, reliability issues were observed, which can be addressed by using commercial sensors, which have a very high price range. The paper focuses on sensors for soil water content analysis, without discussing other important parameters such as the temperature, pH, relative humidity, and light.

In [18], the authors conducted an extensive literature survey on the implementation of precision agriculture, and it was found that there is no proposed framework to aid in the implementation of PA (precision agriculture). It was also observed that remote sensors were among the topmost technologies used in PA. The authors also pointed out that the implementations of PA in different studies have mostly been carried out on five different crops, including corn, wheat, soyabean, sugarcane, grapes, and cotton. None of the implementation-related papers consider saffron and the sensor selection criteria and configuration used for its implementation.

In [19], sensors are explored as the most important tool for the implementation of IoT. A wide variety of agricultural sensors, based on their applications, are presented. The sensors were classified as optical sensors, electromagnetic sensors, electrochemical sensors, location sensors, acoustic sensors, and airflow sensors. It was also found that agricultural sensors enhance the productivity of agriculture. Some of the challenges faced in the implementation of sensors in IoT networks include their customization, uninterrupted Wi-Fi connectivity, the handling of errors and faults, and the selection of the right sensors for different environments.

In [20], soil moisture sensors were used to monitor the moisture content of soil, and humidity measurements were performed using a DHT22 sensor in a natural medium, and the supply of water, if required, was automated through a web-based application. The authors did not discuss other important parameters for growth using the system. The results obtained were not defined for any particular crop, as the water and humidity requirements of each crop are different.

The detailed analysis of the different studies carried out by researchers in the field of PA can be summarized in Table 1, along with their features and the shortcomings of the presented study.

**Table 1:** Various Sensors Considered by Researchers in Precision Agriculture.

Ref/ Year	Sensors Used	Medium for Cultivation	Crop Used for Research	Implementation	Application Domain
[20]/ 2022	pH sensor Electrical conductivity sensor Water level sensor	Greenhouse	Tomatoes	Simulation and hardware-based	Monitoring
[21]/ 2020	DHT11 sensor LM35 soil temperature sensor pH sensor Soil moisture sensor MQ135 CO_2_ gas sensor	Artificial	Cucumber	Simulation and hardware-based	Controlling
[22]/ 2020	Thermal sensors	Natural	Onion	Hardware-based	Monitoring
[23]/ 2016	PIR sensors Heat sensor URD sensor	Natural	Staple Crops	Simulation-based	Monitoring
[24]/ 2020	Soil moisture sensor	Artificial	None	Simulation and hardware-based	Monitoring
[25]/ 2020	Soil moisture sensor Salinity sensor pH sensor Electromagnetic sensor	Natural	None	Simulation and hardware-based	Land Suitability assessment
[26]/ 2019	Humidity sensor Soil temperature sensor Temperature sensor Luminosity sensor	Artificial	Argula	Hardware-based	Monitoring
[27]/ 2018	Optical sensors Electrochemical sensors Airflow sensors Mechanical sensors Location sensors	Both	All Crops	Survey-based	Monitoring
[28]/ 2019	Different sensors	Natural	All Crops	Simulation-based	Monitoring
[29]/ 2019	Soil sensor Temperature sensor pH sensor Humidity sensor	Natural	Turmeric	Hardware-based	Controlling
[30]/ 2018	Humidity sensor Temperature sensor Luminosity sensor Water consumption sensor	Greenhouse	Cherry Tomatoes	Hardware-based	Tracking and monitoring
[31]/ 2019	Temperature sensor Humidity sensor Soil moisture sensor	Natural and Greenhouse	Mixed crops	Hardware-based	Controlling
[32]/ 2019	Soil moisture sensor Temperature and Humidity sensor	Greenhouse	Potato	Hardware-based	Monitoring
[33]/ 2018	Temperature sensor Soil moisture sensor Humidity sensor	Natural	Banana	Simulator-based	Monitoring
[34]/ 2018	Temperature sensor Humidity sensor	Natural	All Crops	Hardware-based	Monitoring
[35]/ 2019	Many Sensor	Natural	Vineyard	Hardware- and Simulator-based	Monitoring
[36]/ 2018	pH sensor Temperature sensor Humidity sensor CO_2_ concentration sensor	Greenhouse	Strawberry	Hardware-based	Monitoring
[37]/ 2019	Soil moisture sensor Luminosity sensor Water level sensor Temperature sensor	Natural	Name of the crop not revealed	Hardware-based	Monitoring
[38]/ 2019	Temperature sensor Water level sensor	Natural	Soyabean	Hardware-based	Monitoring
[39]/ 2019	Humidity sensor Light sensor CO_2_ concentration sensor Temperature sensor Windspeed sensor Direction sensor Camera sensor	Natural	Saffron	Proposed model	Monitoring

As per the survey conducted, it was found that there are different sensors for the same parameter on the market. It is always difficult to choose the best sensor for the research and project work.

### 2.2. Sensors Used in Precision Farming

A thorough investigation was conducted, and the sensors most commonly used for the parameters related to saffron cultivation were identified [40,41,42,43,44,45,46,47,48,49,50,51,52,53,54,55]. The results of the study, depicting various parameters and their values, are summarized in Table 2. A comparison between the different sensors and their counterparts is also given in Table 2.

**Table 2:** Sensors Available and Their Comparison.

Luminosity	Water Flow& Turbidity	Corm Weight	Ph	Temperature	Agronomical Variable
TSL2561 TCS3472	Seed Studio Grove TDS Sensor Water Flow Sensor YF5201	Load cell Piezoresistive sensors	DFRobot Gravity Analog pH Sensor EZO TM class embedded pH circuit Industrial Grade Ph Sensor PH2.0 Interface	LM35 DTH-11 DHT22 AM2302 SHT71	**Sensors Available**
2.7 V to 3.6 V 3 V to 5 V	3.3 V to 5 V 5 Vto 29 V	5V to 12 V 0V to 5 V	3.3 V to 5 V 3.3 V to 5 V 2 V to 5 V	−0.2 V to 35 V 3 V to 5 V 3 V to 5 V 3.3 V to 5.5 V 2.4 V to 5.5 V	**Operating Voltage**
75%to 80% High	±3 L/min ±2l L/min	0.05% 0.02%	±0.1 pH ±0.02 pH ±0.01 pH	±1 °C ±2 °C and 5% ±5 °C and ±2–5% ±2% RH 0.5 °C ±0.4 °C 0.1	**Precision**
$7.75 $8.81	$37.17 $6.29	$6.16 $8.81	$62.90 $44.03 $25.16	$0.68 $5.03 $6.79 $11.32 $70.45	**Cost**
Highly sensitive Highly sensitive	±5% ±3%	3 mV 19 HZ/KPa	High 0.02 High 0.02 High 0.02	-- -- -- 0.1%RHand 0–1 °C 0.04–0.01 °C	**Sensitivity**
1.25 mm × 1.75 mm × 3.1 mm 2 mm × 2.4 mm × 2.1 mm	20 cm × 40 cm 1.2 mm diameter	55.2 mm × 12.78 mm × 12.7 mm 400 µm × 400 µm × 10 µm	42 mm × 32 mm × 1.66 mm 13.9 mm × 20.16 mm × 80.38 mm 50 mm × 47 mm × 16 mm	4.699 mm × 4.699 mm 11 × 8.26 × 0.62 inches 14 mm × 18 mm × 5.5 mm 14 mm × 18mm × 5.5 mm 13.5 mm × 5.08 mm × 3.1 mm	**Dimensions**
0.1to 40,000 Lux 1–3,800,000 Lux	0 to 1000 ppm 1–5 L/min	0 kg to 5 kg 1.5 Psi to 100 Psi	0 to 14 0.01 to 14.00 0 to 14	−55 °C to 150 °C 0 °C to 50 °C −40 °C to +125 °C (0 to 100% RH) −40 °C to 123.8 °C	**Range**
8–11 h	80 m/h	5.5 gm to 10 gm	6 to 6.4	16 °C to 27 °C 60% to 80%	**Range required for artificial cultivation**
Very Less Less	Very Less Less	Very Less Less	Less Very Less Very Less	±0.2 °C ±0.5 °C ±0.2 °C	**Error value**
>30 min >1 h	<3 min <30 L/min>	<8.5 HZ <6 HZ	2 min 10 min <1 min	0.1 Hz 1 Hz 0.5 Hz 0.5 Hz 0.2 Hz	**Sampling rate**

## 3. Problem Formulation

The sensors selected for artificial cultivation and natural cultivation differ greatly. The presence of different sensors also creates a great deal of confusion due to absence of a proper standard for consideration. It was also observed that there is no sensor-based system for artificial saffron cultivation guiding the sensor selection procedure. Thus, there is a great need to define the sensor configuration for saffron cultivation in an artificial medium. Hence, using the proposed novelty-based model, we define all the sensors required for saffron cultivation, along with their details, configurations, and features for an optimal yield and quality of the saffron. Moreover, it was also observed that, due to the lack of research papers related to the IoT and soilless saffron cultivation, different research questions have not been addressed in any of the papers. These questions are:

RQ1 What is the percentage increase in crops cultivated using artificial methods and IoT?
RQ2 What criteria are used to select sensors for soilless saffron cultivation?
RQ3 What are the most frequently used sensors in IoT-based artificial saffron cultivation?
RQ4 What is the percentage use of sensors for different practices related to saffron cultivation?
RQ5 What is the production increase in major crops of the world and saffron after the use of the IoT?

Due to the immense importance of the above-stated research questions, a thorough investigation of the literature was performed to obtain answers to these questions. For new researchers aiming to cultivate saffron in an artificial medium using the IoT and sensors, these questions are of the utmost importance, as none of the existing studies in the literature provide answers to them. The responses to these questions are discussed in Section 5.

## 4. Proposed System Model

The proposed system model was designed for the artificial cultivation of saffron in a water medium using hydroponic the NFT (nutrient film technique). The system model is implemented using solar energy to minimize its energy consumption and make the system cost-effective. The model had already been designed, and the saffron corms were in the incubation period. The hardware components used, their configuration, and the block diagram are described in the following subsections.

### 4.1. Hardware Setup and Design

On the basis of the range of values that are sensed for saffron cultivation, the sensors chosen were DHT-11 for the temperature and humidity control, an industrial-grade Ph sensor, PH2.0 Interface, for the pH, a load cell sensor for the weight monitoring of the corms, the Seed Studio Grove TDS Sensor for the water flow and turbidity, and TSL2561 for the luminosity. Water flow is an important parameter in a hydroponic system and can be obtained using the fluid dynamic expressions given in Equations (4)–(8). Although the other sensors of temperature and humidity offered a greater precision and wider range than DTH-11, due to its cost and the fact that the range to be monitored and controlled did not exceed 27 °C for the temperature and 80% for the humidity, DHT-11 was the best choice. Similarly, for the pH, the industrial-grade Ph sensor pH 2.0 Interface was chosen because it is economical, offers a higher precision and sampling rate and lower error rates, and has smaller dimensions as compared to the other existing options. In our setup, the load cell is used to calculate the corms’ weight, as it offers an accuracy of 0.02% and has lower sampling rates. Although the cost of the load sensor is slightly higher than its counterpart, due to the precision required for measuring the corm weight and the fact that the corm weight is a parameter of very high importance, this sensor was chosen. To control the luminosity, TSL2561 was used due to its high accuracy and low cost.

**Figure 4:**
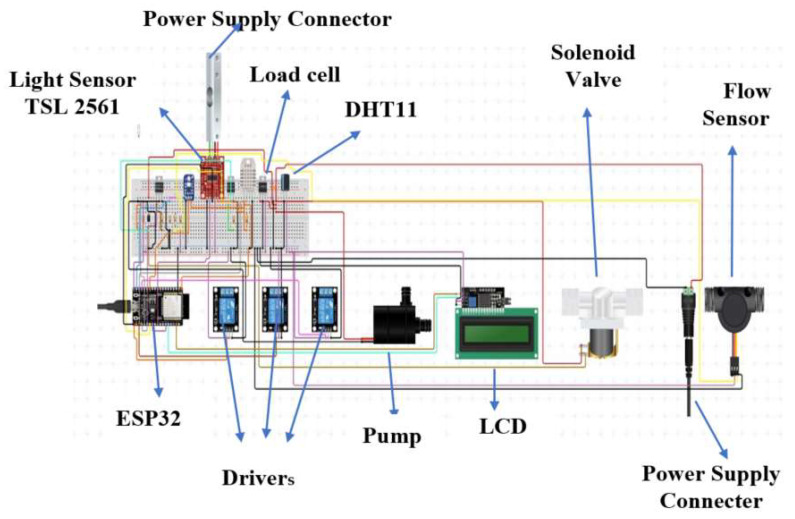
Simulation Circuit Diagram Showing the Sensors and Devices Connected to the Board.

The simulation environment used for configuring the sensors and all the electronic devices before installation was Node Red. Node Red is a device-based flow editor which ensures the compatibility of all the sensors, enabling them to operate together, before installation. Keeping the cost and requirements in view, the microcontroller used was ESP32 WROOM 32, which is a Wi-Fi and Bluetooth microcontroller unit module with inbuilt networking capabilities and high scalability options. The recommended operating voltage and temperature for ESP32 WROOM 32 are 2.7 V to 3.6 V and −40 °C to 85 °C, which are in line with our requirements. The designed circuit diagram, as shown in Figure 4, consists of different sensors and hardware components connected to the microcontroller, which can be listed as follows:

DHT11 humidity sensor
Industrial-grade Ph sensor PH2.0 Interface
Seed Studio Grove TDS sensor
Water flow sensor
Load cell sensor
Servo Motor 9 g
Relay 12 V/5 A
LED light white
LCD 16 × 12
Solar panel
Battery
Heater 1000 W

The connections between the hardware devices and the sensors can be seen in Figure 4.

ESP32 WROOM 32 is a dual-core, low-power-consuming microcontroller with a very reasonable cost. It has integrated Wi-Fi and Bluetooth. The flash memory in this MCU is 4 MiB, with a RAM equal to 320 KiB and ROM equal to 448 KiB. This MCU is programmable in languages such as Visual Studio Code, Micro Python, etc. It is very secure due to its use of flash encryption and WPA encryption standards. It consists of 48 pins, and in the proposed cultivation model, all the sensors and devices are connected to different pins to ensure the automated functioning of the sensors and hardware devices. To measure the humidity and temperature, two DHT11 sensors are deployed, both of which are connected to ESP32. One of the two sensors measures the temperature outside the cultivation medium, and the other is responsible for measuring the outside temperature. In this way, both the temperatures can be compared, and actions, such as turning on the fan or heater, can be controlled, keeping the temperature in the optimal range. The sensor used for measuring the corm weight is a load cell sensor. It has an operating voltage of 5 V to 12 V, with a precision value of 0.05%, making it ideal for our hydroponic system. The most effective parameters for enhancing the saffron yield and quality are the corm size. Hence, this requires great accuracy in terms of the measurements, which can be achieved by using the load cell sensor.

Since the optimal pH value to be maintained for the nutrient solution used is 6–6.4, the industrial-grade Ph sensor, pH 2.0 Interface, is used. This sensor not only senses the pH value in real time but also controls it by altering the flow rate of the nutrient solution. It also offers precision values as low as ±0.01 pH, making it ideal for a hydroponic system.

The Seed Studio Grove TDS sensor is used to monitor the water quality and presence of unwanted pathogens or substances that are harmful to a hydroponic system. The operating voltage of this sensor, measured precision of the range of values, and other details are given in Table 2. The water flow sensor used in the hydroponic system is YF5201. This sensor has an operating range of up to 80 mL/h, making it ideal for a hydroponic system. The Servo Motor 9g is a small actuator controlled by ESP 32, responsible for controlling the pumping of the water from the nutrient tank of the hydroponic system into the growth chamber, which is placed on its top. It requires a DC supply of 4.8 V to 6 V, with a duty cycle of 1.5 ms. It functions with the flow sensor to control the flow rate of the nutrient solution for optimal results. The system is also equipped with three relays operating at 12 V/5 A.

Relays are electromechanical switches which control AC devices using DC power. In the proposed system, relays are used for controlling the heater, battery, pump, and solenoid valve. The solenoid valve in the system is responsible for regulating the water flow and mixing of nutrient solutions in the water. The proposed system also consists of an LED emitting white light, as the saffron plant needs light for 8 h a day for its optimal growth and flowering. Since the corms are sown in August, there are decreasing temperatures that are not optimal for saffron cultivation in many places. This problem is solved by using the 1000 W heater, operated by one of the relays to control the temperature for the optimal growth of the plants. A solar panel and a battery are also used, which are connected to another MCU, serving as sustainable power sources and ensuring the operation of the designed hydroponic system using the dual modes of the power supply. All the readings from the different sensors are indicated by a 16*12 LCD, which is controlled by both the MCU’s, displaying data from time to time.

### 4.2. Block Diagram and Working

**Figure 5:**
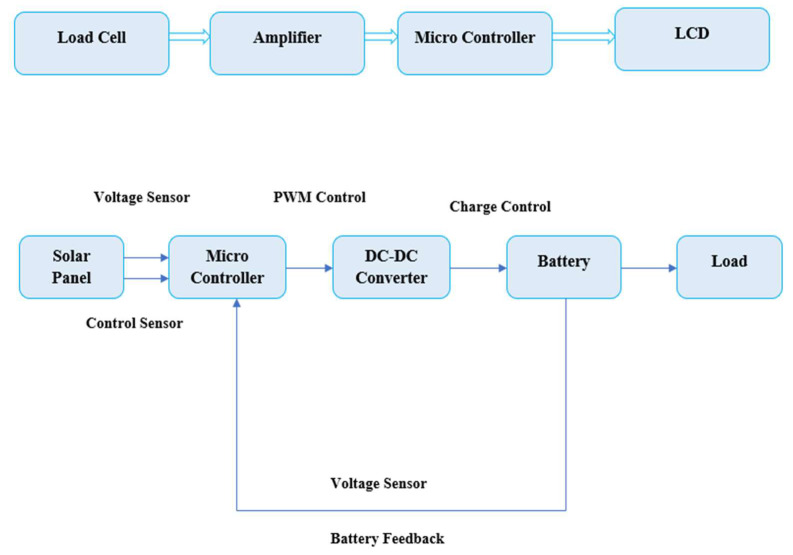
Block Diagram Showing the Solar Panel and Battery Storage of the System.

Saffron cultivation in the proposed system is performed using hydroponics. This refers to the cultivation of plants in a soilless medium using water and the necessary nutrients. There are many methods of hydroponic cultivation, and the method used in the given system is NFT, which we selected to make the system reliable and eliminate water wastage. The system model consists of two ESP32 units. In one of the subsystems, as shown in Figure 5, the MCU is connected to the solar panel. The solar panels are equipped with voltage and control sensors ensuring the conversion of the DC power into AC, which is further lowered to suit the sensors and other devices using PWM (pulse width modulation) through a DC-DC controller. The derived power is stored in the battery, which can be used to operate part of the hydroponic system in times of power failure.

**Figure 6:**
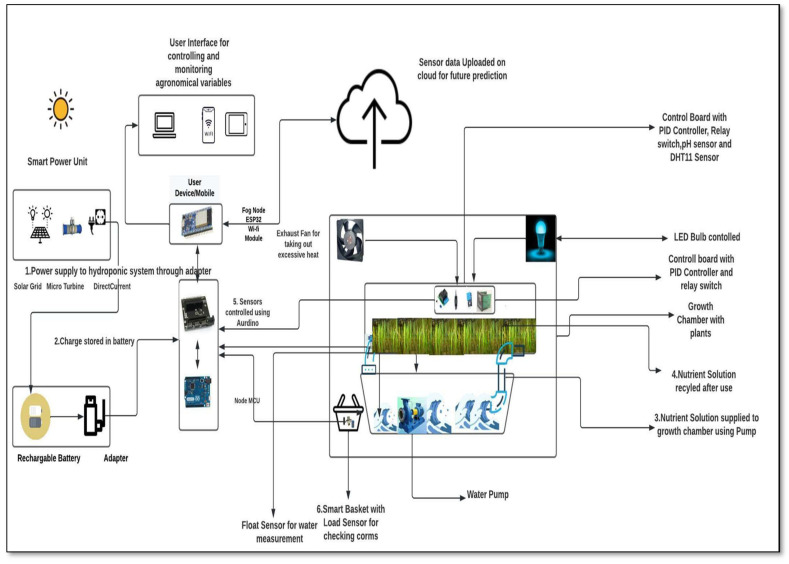
Automated Saffron Cultivation System Using a Hydroponic Setup.

The proposed smart hydroponic system, shown in Figure 6, can be used to maximize the efficiency parameters, such as the yield and quality. This model shows the cultivation of saffron in a soilless medium based on the minimization of the cost and use of renewable sources of energy. It makes use of solar energy, enabling the use of cost-effective alternatives and the system’s use in areas where electricity is scarce [55]. NFT is used for the water and nutrient uptake, enabling the recycling of the water. The components of the system include its dual power supply, plant growth chamber, smart corm weight basket, nutrient tank, interface, and cloud storage for the data. Initially, the user selects the power supply mode, and the minerals and nutrients in the tank are transported to the growing plants using the pump.

The synchronization and data sharing between the units can be seen in Figure 6. Different agronomical variables such as the temperature, corm size, pH, mineral concentration, and electroconductivity of the solution are analyzed at short intervals by the sensors deployed, and the data are stored on the cloud, shared on a real-time basis on the mobile and laptop interfaces, and used for the predictive analysis. The different minerals required by the saffron for cultivation are mixed with water in the required proportions and then pumped into the growth chamber [56]. The required mineral concentration provided to the growing plants is identified using Equations (1)–(3).

After checking the weight of the corms using smart basket, the mass and size (>10 gm) parameters are used to sort the high-quality corms to be used for the sowing [57]. The selected corms are treated with a Tiabendazole solution for protection against fungal infections [58]. The nutrients mixed in oxygenated water are continuously circulated at the desired flow rate using the pump and air stone. The desired space between two corms is calculated to ensure the maximum productivity [59]. The flowering of saffron plants in hydroponic medium is expected to occur after 90 days. The stigmas collected from the saffron flowers are dried (moisture < 30 percent) before being made available on the market. Table 3 presents a list of suitable, effective nutrients and different parameters, with the optimized values required for the soilless cultivation of saffron [56,57,58,59].

One of the major challenges when using a hydroponic system is maintaining the ion concentration of the nutrients used in the solution of the hydroponic system. To avoid nutrient imbalance, the electroconductivity of the system needs to be maintained [59,60,61]. The total ion concentration can be controlled while minimizing nutrient imbalance through the injection of the nutrient solution. For the hydroponic system to operate efficiently, the nutrient mixing procedure needs to be understood. The nutrients in the nutrient solution are measured to detect changes in the nutrient tank, and then the process of mixing the nutrients is conducted at intervals [62]. This can be shown in the form of Equation (1), as:

(1) $$C_{t}EC_{t}=C_{c}EC_{c}+bUU=\frac{C_{t}EC_{t}-C_{c}EC_{c}}{b}$$

where *C_t_* is the maximum achievable capacity of the solution in the tank; *EC_t_* is the target electroconductivity value in (dS m^−1^) to be achieved; *C_c_* and *EC_c_* are the current volume of the nutrient-rich solution and current electroconductivity value of the nutrient tank, respectively; *U* represents the nutrients absorbed by the plants in milli; and *b* is the empirical coefficient for the conversion of the *EC* and concentration of the salt. A value of the coefficient of *b* = 9.819 is considered suitable for hydroponic nutrient solutions [63], while an *EC_t_* value range of 0.8–4.0 dSm^−1^ can be considered suitable. Using Equation (1), the amount of nutrient solution required, can be calculated by using Equation (2):

(2) $$C_{t}EC_{t}=C_{C}EC_{C}+C_{w}EC_{w}+C_{stk}EC_{stk}\;\;C_{stk}=\frac{C_{t}EC_{t}-C_{C}EC_{C}-C_{w}EC_{w}}{EC_{stk}}\;$$

where *C_w_* is the water required; *EC_w_* is the electroconductivity of the water; *C_stk_* is the amount of nutrients required in the solution; and *EC_stk_* is the conversion factor of the milli unit concentration of the solution to *EC*. *C_t_* should be equal to the sum of *C_c_*, *C_w_*, and *C_stk_*. Therefore, the following equation (Equation (3)) can be derived using this relationship:

(3) $$C_{stk}=\frac{C_{t}EC_{t}-C_{C}EC_{C}-EC_{w}\left(C_{t}-C_{C}\right)}{EC_{stk}-EC_{w}}$$

After calculating *C_stk_* and *C_w_*, the obtained values are provided to the sensors and microcontroller as the input values for the operation of the pumps and solenoids valves.

The flow rate (in Table 3) of the water in a hydroponic system is yet another important parameter, which depends on the diameter of the pipe used and is governed by the equations of the fluid dynamics related to continuity, energy, and momentum. It was observed that for a flow pipe with a diameter of 9.5 mm, the flow rate was estimated to be 1.5 L min^−1^ [64]. The computational fluid dynamics of the nutrient solution can be computed using Equations (4)–(8):

(4) $$\mathrm{Continuity}\;\;\nabla \cdot \left(\rho \vec{u}\right)=0$$

(5) $$\mathrm{Momentum}\;\;\nabla \cdot \left(\rho \vec{u}\vec{u}\right)=-\nabla_{i}^{p+\nabla }\cdot \left(\bar{\bar{\tau }}\right)\rho_{\vec{g}}$$

(6) $$\bar{\bar{\tau }}=\left[\left(\left(\nabla \vec{u}\right)+{\left(\nabla \vec{u}\right)}^{T}\right)-\frac{2}{3}\nabla \cdot \vec{u}\bar{\bar{I}}\right]$$

(7) $$\mathrm{Energy}\;\;\;\;\;\nabla \cdot \left(\rho \vec{u}H\right)=\nabla \cdot \left(\frac{k_{t}}{C_{P}}\nabla H\right)+S_{h}$$

(8) $$H=\int_{T_{0}}^{T}C_{p}dT$$

where $\vec{u}$ is the velocity vector, and the density and pressure are represented by *ρ* and *p*, respectively. *S_h_*, *C_p_*, = I, = τ and *T* are the source term, specific heat, identity matrix, stress tensor, and temperature.

*µ*, *H*, *k_t_* and $\vec{g}$ represent the viscosity, enthalpy, thermal conductivity, and gravitational acceleration, respectively.

The flow patterns in fluid mechanics can be predicted by using Reynold’s number (Re). It is used in simulations, as well as experimental or empirical estimations [65,66]. The Re is defined by Equation (9) as:

(9) Re=ρνDHμ

where *µ* denotes the dynamic viscosity of the fluid (kg ms^−1^), *D_H_* represents the hydraulic diameter (m), *v* is the velocity of the fluid (m s^−1^), and *ρ* denotes the density of the fluid (kg m^−3^).

### 4.3. Hardware Setup for the Hydroponic Cultivation of Saffron

**Figure 7:**
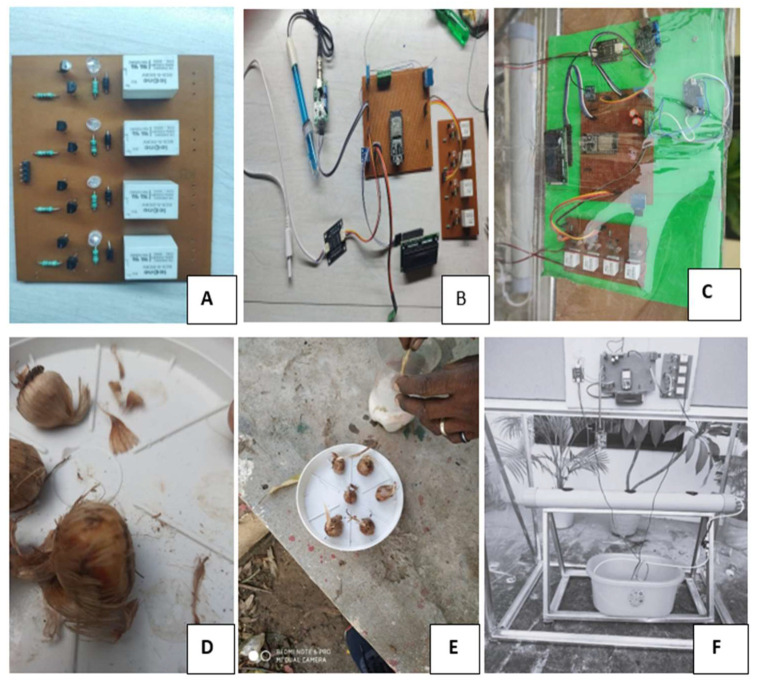
Step-Wise Setup of the Hydroponic System: (**A**) Components Used for the Circuit; (**B**) Intermediate Stage; (**C**) Final Circuit diagram; (**D**) Identification of Saffron Corms for Sowing; (**E**) Treatment of Corms with Fungicide Before Sowing; (**F**) Placing the Corms in the Hydroponic Solution.

The setup designed for the cultivation of saffron consists of the hardware components already explained in Figure 4. After designing the circuit and joining the components, the best-quality corms, on the basis of their weight (>8 gm), were chosen. The chosen corms were treated with 0.2% tiabendazole and dried in shade for one day. After this, the corms were sown in the hydroponic system and were constantly monitored for their growth and the control of agronomical variables. The essential nutrients were supplied to the plant through the pump using the nutrient solution, which was continuously recycled [67,68]. The corms were in their very initial development phase, and the effect of the controlled environment on the yield, quality, and time taken to flower will be covered in future studies. The detailed step-by-step procedure of the designed the hardware system for cultivation is shown in Figure 7.

## 5. Results and Discussion

### 5.1. Analysis of the Research Problems

This section provides an analysis of the critical factors needed for the artificial cultivation of saffron, such as the criteria for selecting the sensors, the most commonly used sensors, and the application of sensors for different practices related to saffron cultivation. The analysis and reports are based on the existing RQs in Section 3:

RQ1: What is the percentage increase in crops cultivated using artificial methods and the IoT?

**Figure 8:**
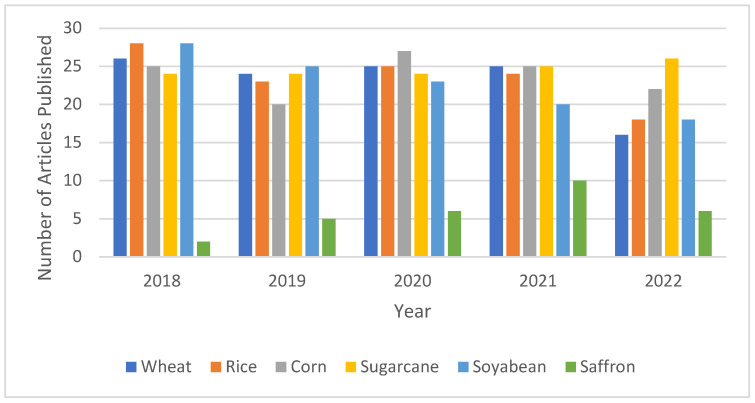
Publications Per Year for the Leading Crops of World.

After studying the literature, it was observed that the major crops of the world, as per their production, are wheat, rice, corn, soyabean, and sugarcane [65,66]. Out of these crops, the percentage of the yield produced by using IoT and other technologies, based on the literature published from the year 2018 onwards, has increased in the case of different major crops and can be shown in the form of a graph, as shown in Figure 8. It has also been observed that the research carried out on saffron is limited to natural ecosystems, without realizing the economic potential of artificial saffron cultivation, and the publications on this topic are limited.

RQ2: What criteria are used to select sensors for soilless saffron cultivation?

The second motive for conducting this research is the challenge of selecting sensors out of a wide range based on their suitability to the environment. For this purpose, an in-depth literature review was conducted. The selection of sensors depends on different factors, such as the requirements for the application, cost, error percentage, size, weight, etc. [44,45,46,47,48,49,50]. Out of all the different selection parameters discussed by researchers, the major ones identified are listed in Table 4, given below, with their descriptions.

Based on the above-described selection of the parameters, the different sensors widely used in PA were considered for the use case of saffron cultivation. To select a particular type of sensor, a detailed step-by-step comparison was followed:

- First of all, the application and the environment in which the sensor was destined to be used and the range of the values which were intended to be sensed were defined. The range of the values was identified by studying various research articles related to the agronomical variables used for saffron cultivation [51,52,53,54,55,56].
- The major variables to be controlled and monitored were found to be the temperature, humidity, pH, water flow, weight of the corm, and luminosity. The sensors available for each variable were compared on the basis of the selection parameters and suitable nutrients given in Table 3.

RQ3 What are the most frequently used sensors in IoT-based artificial saffron cultivation?

**Figure 9:**
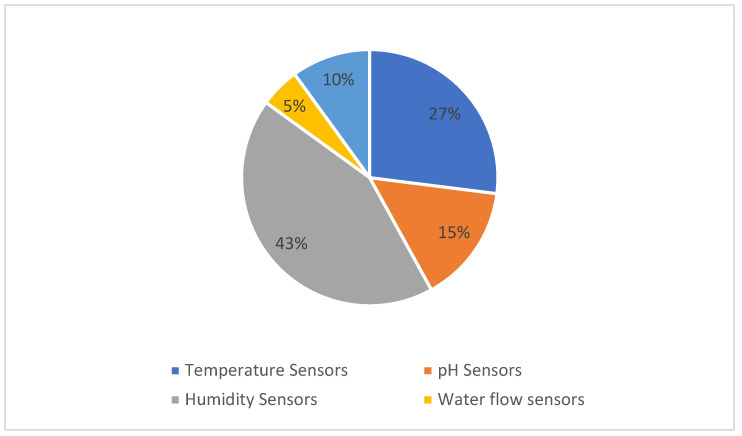
Sensors Used in Precision Agriculture.

In addition to this, a few researchers have focused on the use of sensors for the control of agronomical variables, with most of them focusing on monitoring. Based on the literature survey [41,42,43], the top five most commonly used agricultural sensors can be represented as given in Figure 9. In Figure 9, it can clearly be seen that the sensors most frequently used in agriculture are given as:

RQ4 What is the percentage use of sensors for different practices related to saffron cultivation?

**Figure 10:**
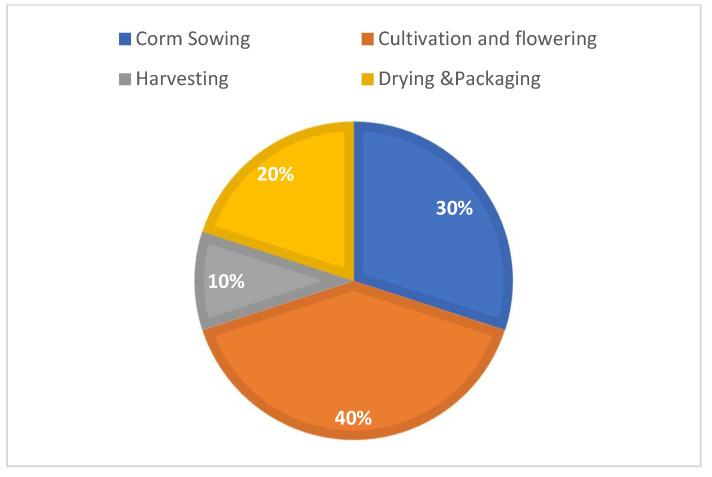
Percentage Use of Sensors in Saffron Cultivation Practices.

The major practices related to saffron cultivation are corm sowing, flowering, harvesting, and drying and packaging [51,52,53,54]. The percentage-wise distribution of the use of IoT in all these practices can be given as shown in Figure 10. The maximum use values of the sensors and IoT were found in papers related to cultivation and flowering, followed by corm sowing and drying, with only 10% of the articles related to harvesting, which is the most difficult and laborious phase of saffron cultivation.

RQ5 What is the production increase in major crops of world and saffron after the use of IoT?

**Figure 11:**
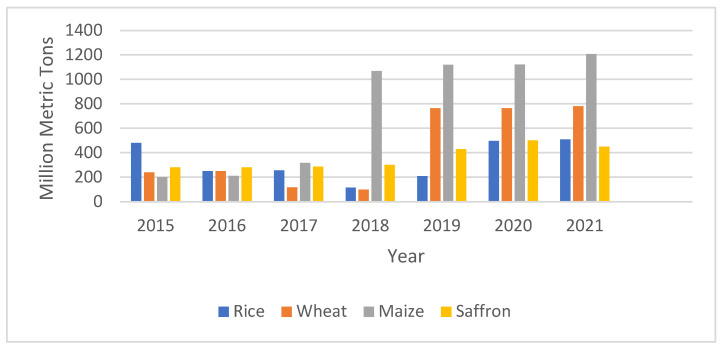
Production of Crops Over Previous Years.

As per the reports and different research articles, the production of crops such as rice, wheat, and maize has greatly increased through the use of IoT and smart farming. The production of rice using IoT was 480 million metric tons in 2015, which increased to 510 million metric tons in 2021.The impacts of the IoT and sensors on corn cultivation have increased its production significantly, with 1200 million metric tons in 2021. However, due to the practice of area-specific cultivation and the limited research on saffron and the use of IoT in saffron cultivation, the IoT has only minimally benefited saffron production, increasing the production from 279 million metric tons to 450 million metric tons, primarily in Iran [61,62]. From the data given in Figure 11, we can see that there is a low production of saffron as compared to other crops in all the years. Hence, there is great scope for the use of new technologies such as the IoT to provide greater economic benefits.

### 5.2. Results from Hardware Setup

**Figure 12:**
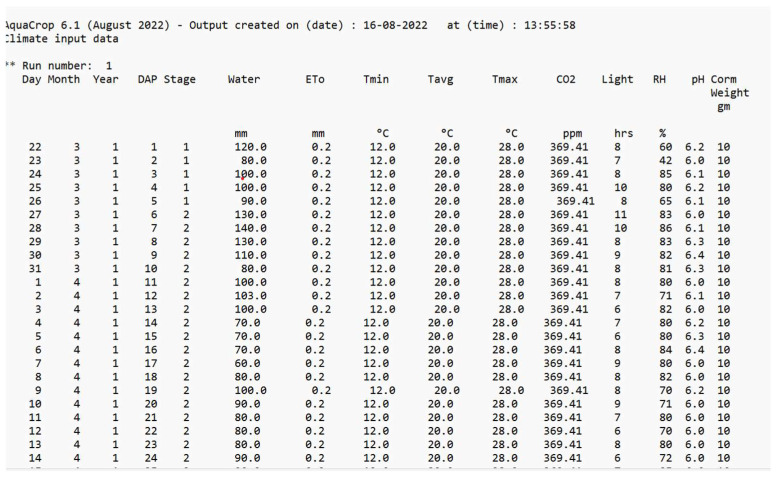
Input Parameters for the Climate File.

One of the most important growth models for the simulation of the yield and biomass of crops growing in hydroponic systems is AquaCrop. The reason for considering this model is that only a few studies have been conducted on the parametrization of saffron crops. AquaCrop simulates the yield response to water in the case of herbaceous crops and is particularly well-suited to conditions where water is a key limiting factor in crop production. The long growing cycle of the crop makes the simulation of its growth parameters difficult [69]. The dataset considered in this study is based on the values obtained from the sensors of the hardware model. The results obtained indicated that the yield and biomass can be considerably improved in a hydroponic medium, with a reduction in the flowering time, better water productivity, and less time required for cultivation. The important agronomical variables affecting the growth, which are controlled and monitored through the hardware model, are used as inputs to the AquaCrop model. Figure 12 shows a screenshot of the input parameters considered for AquaCrop.

**Figure 13:**
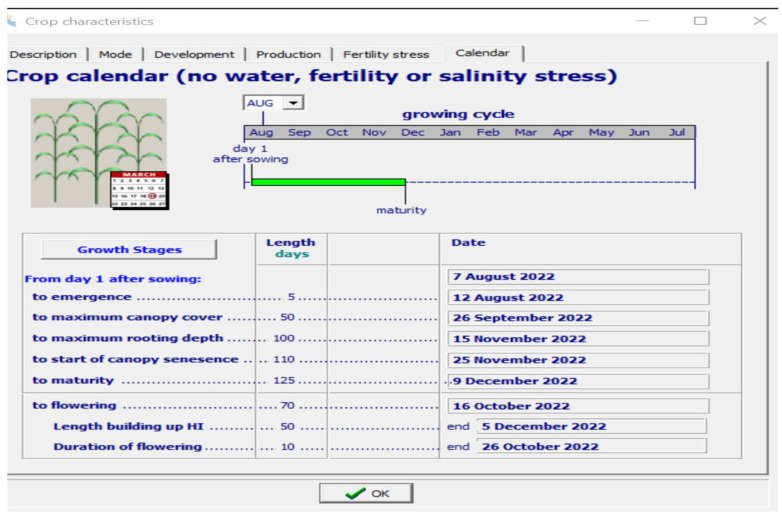
Growth Stages and Timeline for Saffron.

**Figure 14:**
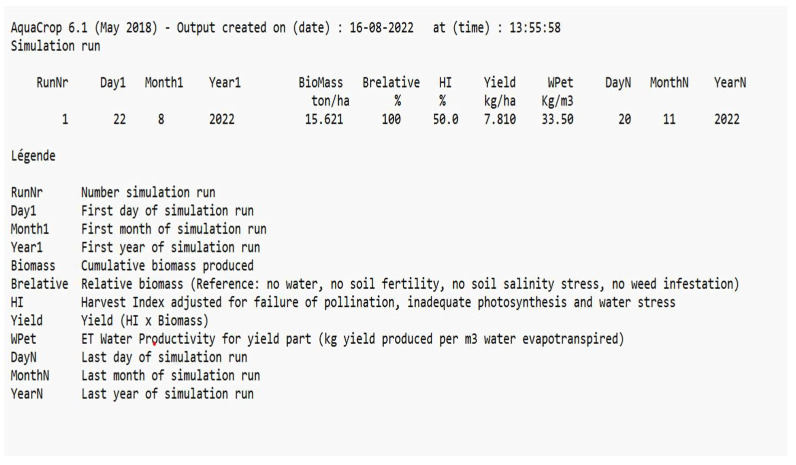
Screenshot of Output Parameters after the Simulation Run.

The results of the simulation are shown in Figure 13 and Figure 14. As shown in Figure 12, the timeline for the cultivation of the saffron is considerably reduced in comparison to its natural cultivation, which takes around 280 days for its completion [70]. The maximum canopy cover, which gives the percentage of the maximum ground area covered by native vegetation, is predicted to be achieved after only 35 days of sowing. The flowering also occurs early in this case.

The water productivity is used to evaluate the performance. It refers to the biomass produced per meter of water lost in evapotranspiration and should be as high as possible. It measures the performance and efficiency of water-based models. The water productivity obtained in the case of the hydroponic model was 2.27 kg/m^3^, as compared to 1.9 kg/m^3^. The biomass value, which gives the living tissue mass expressed in mass per unit area, for the hydroponic medium was obtained as 11.780 ton/ha, as compared to 11 ton/ha in the case of natural cultivation. The value of the harvest index, which gives the total weight of the stigmas divided by the total above-ground biomass, was obtained as 50% [71]. Using the biomass and harvest index, the yield can be calculated using Equation (10) as:

(10) y=B×HI

**Figure 15:**
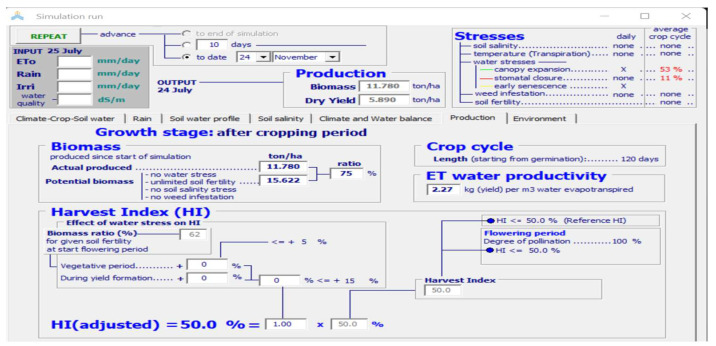
Water Productivity and Harvest Index.

The value of the yield obtained through hydroponic cultivation was 7.8 kg/ha, and it was 4.7 kg/ha in the case of natural cultivation [71,72] as shown in Figure 15.

## 6. Conclusions and Future Work

The world population is increasing every year. There is a need for the development of artificial cultivation methods for agriculture, as the land under agriculture is also declining due to the increase in populations and urbanization. Artificial cultivation methods such as hydroponics can realize the full potential by using the internet of things, using sensors as the backbone. Due to the increase in the production of sensors, there is a great need to develop a standard sensor selection procedure. The crux of the problem, based on the literature studied, is that most research articles on PA are related to major crops of the world, such as corn, soyabean, and wheat. It was also seen that the sensors used in PA focus mostly on five parameters, which include the temperature, humidity, water flow, and light. It was also seen that the sensors used in PA are mostly used in natural media for the monitoring of values. The important factors to be considered in sensor selection for a particular application should be based on the nature of the application, accuracy, response time, and range of values to be monitored and controlled. Following the literature study, a system model for the artificial cultivation of saffron was proposed. As saffron is the most expensive spice in the world, it offers great economic benefits and has a vast array of applications. The number of research articles dedicated to artificial saffron cultivation and the sensors used is very small, which makes this an interesting topic of research. The information related to the sensors and the selection procedure for saffron cultivation in hydroponic media was also discussed.

The hardware setup and the framework for saffron cultivation using hydroponics were proposed. The important factors in a hydroponic system for saffron cultivation, such as the flow rate and electroconductivity, were explained using equations. The sensor selection procedure followed and the suitable sensors on the basis of factors such as the sampling rate, cost, precision, and operating range for hydroponically growing saffron were also discussed. The hardware model was validated using the AquaCrop model to predict output parameters such as the yield, biomass, water productivity, and harvest index. The values obtained indicate better results as compared to those of natural cultivation, which is time-consuming.

The future scope of this study will involve a comparison of the predicted results obtained using the AquaCrop model with the actual results. The saffron corms already sown in the automated hydroponic setup are being monitored and controlled in different stages. In future work, a comparison of the results with the present state of the art will be considered in light of other important parameters for sustainable cultivation, such as carbon emissions. The accuracy of the data received through the cloud using an app will also be studied within the future scope of this study. We will explore parameters such as the stigma size and quality, number of flowers per plant, and cost optimization with respect to the existing state of the art and naturally growing saffron.

## Figures and Tables

**Table 3:** Suitability of Effective Nutrients in Saffron Cultivation.

S No.	Parameter	Value
1	pH	6–6.4
2	Copper	<5 ppm
3	Water requirement	400 mm
4	Temperature	23–27 °C first (90–150 days) then 25 °C
5	Incubation temperature	23 °C
6	Relative humidity	60–85%
7	Light	8–11 h in hydro
8	CO_2_	400 ppm
9	Temperature after incubation	17 °C,+−2
10	Relative humidity after incubation	60%
11	Incubation temperature	16–23 °C
12	Flowering temperature	23–27 °C
13	Corm weight	5.7 g, (3.2–3.5 cm diameter)
14	Nutrient solution	Half-strength Hoagland medium
15	Electroconductivity	1100 µs/cm,
16	Flow rate	80 mL/h
Macro-Nutrients(mg L^−1^)		
17	Nitrogen	163.20
18	Phosphorus	34.52
18	Potassium	172.56
20	Calcium	105.11
21	Magnesium, sulfur	33.8, 62.70
Micro-Nutrients (mg L^−1^)		
22	Iron	1.83
23	Boron	0.23
24	Mn	0.27
25	Zinc	0.19
26	Copper	0.12
27	Molybdenum	0.07

**Table 4:** Sensor Selection Criteria in Precision Agriculture.

Selection Parameters	Description
Resolution	It is the smallest measurable change in the values which can be detected.
Range	It includes all the values between the maximum and minimum. It depends on the application values which need to be sensed.
Precision	Closeness of sensor reading to the true value. It should always be high to ensure optimal results.
Cost	The sensor selection should be performed while keeping the cost in view with respect to the application for which the sensor is being used.
Error percentage Response time Dimensions (size and weight)	It is defined as the difference between the measured value and the true value. Ideally, it should always be the minimum. It is defined as the time lag between the input and output and should be low. The selected sensors should always be compact in size and light in weight.
Calibration	The operation of the sensors should be easy and frequent in calibration.
Sensitivity	It is the ratio given by the change in the output to the change in the input and is preferably high. This is directly proportional to the cost.

## Data Availability

Data will be made available on request.
